# Intrinsic Repeated Self-Healing Textiles: Developing Electrospun Fabrics for Enhanced Durability and Stretchability

**DOI:** 10.1021/acsomega.4c09296

**Published:** 2024-12-16

**Authors:** Tse-Yu Lo, Heng-Hsuan Su, Jhih-Hao Ho, Chia-Wei Chang, Huan-Ru Chen, Hsun-Hao Hsu, Kai-Jie Chang, Jiun-Tai Chen

**Affiliations:** † Department of Applied Chemistry, National Yang Ming Chiao Tung University, Hsinchu 300093, Taiwan; ‡ Department of Chemistry, Duke University, Durham, North Carolina 27708, United States; § Center for Emergent Functional Matter Science, National Yang Ming Chiao Tung University, Hsinchu 300093, Taiwan

## Abstract

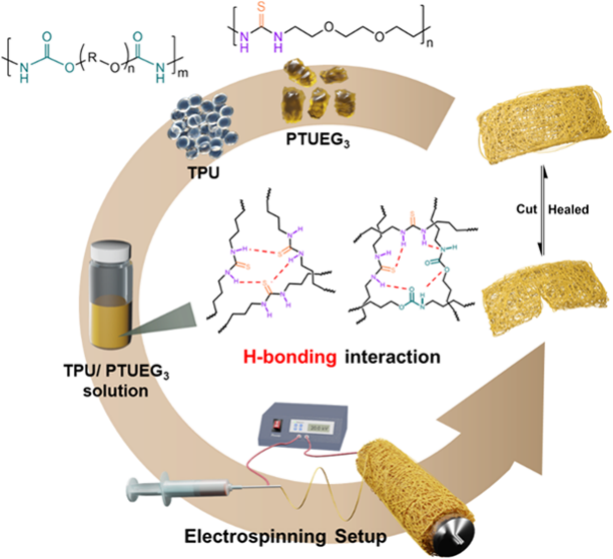

The development of healable polymers represents a significant advancement in materials science, addressing the need for sustainable solutions that can reduce waste and prolong the lifespan of various products. For the development of healable polymer fabrics, however, there are still unsolved issues because of limited healing cycles and poor mechanical properties. In this work, we present intrinsically healable materials for the creation of stretchable, healable fabrics. Specifically, a blend of polyurethane (TPU) and poly(thiourea triethylene glycol) (PTUEG_3_) is fabricated into fabrics utilizing the electrospinning method. The TPU/PTUEG_3_ fabrics demonstrate room-temperature self-healing capabilities over repeated cycles under external forces driven by dynamic hydrogen bonding interactions. Furthermore, their self-healing ability can be enhanced through heating. The tensile tests and differential scanning calorimetry (DSC) indicate that the healing capabilities and mechanical properties can be optimized by adjusting the TPU/PTUEG_3_ weight ratios. This research provides a practical approach for preparing intrinsically healable fabrics with excellent durability and flexibility, offering a sustainable solution to extend the functional life of textiles and reduce environmental impact, thereby promoting environmental sustainability.

## Introduction

Polymeric materials are commonly used nowadays because of their advantages including economical production costs, simple processing methods, and stability. Their remarkable stability, however, presents a challenge, as these materials resist degradation after production, leading to long-term environmental pollution.^1^ The production of polymer materials has been increasing in recent years, contributing to environmental pollution.^2^ Additionally, the low recycling rate exacerbates this issue, which can be attributed to the high costs associated with recycling and reproduction processes, including expenses for collection, sorting, transportation, and reprocessing of recyclable materials.^3,4^ In 2015, around 6300 million metric tons of plastic waste were produced, with 79% ending up in landfills or the natural environment. If current production and waste management patterns persist, it is estimated that by 2050, roughly 12,000 million metric tons of plastic waste will be found in landfills or the natural environment.^5^

For a sustainable society, healable polymeric materials have been receiving more attention in recent years because of their prolonged service life and broad applications.^6,7^ Self-healing polymers can be broadly divided into two categories: extrinsic and intrinsic.^8^ Extrinsic self-healing occurs through the release of dispersed healing agents within a polymer matrix following damage. These agents, typically comprising reactive precursors and catalysts, initiate polymerization reactions and reconstruct cross-linked networks to repair damaged areas. Extrinsic self-healing, however, has the disadvantage of limited healing cycles, restricting the range of applications. Intrinsic healing material systems rely on supramolecular forces,^9^ including hydrogen bonding,^10,11^ host–guest interactions,^12^ and ionic interactions^13,14^ instead of healing agents, thus avoiding issues such as poor stretchability and limited healing cycles. The prominence of H-bonding, the most commonly used noncovalent interaction for self-healing materials, is attributed to its remarkable versatility, directionality, and reversibility.^15^ This interaction plays a crucial role in creating self-healing materials with exceptionally adaptable microstructures. Furthermore, the advantages of intrinsic self-healing polymers extend beyond their application to address some of the limitations associated with extrinsic self-healing polymers.

Despite the prevalence of self-healing polymeric materials in various applications, research on healable polymer fabrics remains scarce and requires further investigation. The ability of textiles to heal could simplify the repair process for damaged fabrics and reduce textile consumption, thereby enhancing environmental sustainability.^16^ In the past decades, the development of healing polymer fabrics has mainly relied on extrinsic methods, which exhibit limited healing cycles.^17^

In this work, we present stretchable, intrinsic healable fabrics consisting of elastomer thermoplastic polyurethane (TPU) and healable poly(thiourea triethylene glycol) (PTUEG_3_). TPU provides stretchability and acts as a spacer, preventing the fibers from diffusing and forming hydrogen bonds with each other, which would disrupt the fibrous morphologies. PTUEG_3_ is healable because it is rich in hydrogen bonds, and its glass transition temperature (*T*_g_) is approximately 20 °C;^18^ the low *T*_g_ facilitates polymer chain diffusion and rearrangement.^19^ The TPU/PTUEG_3_ blends are prepared as fabrics using the electrospinning technique, which is frequently utilized for the production of polymer fibers at the micrometer to nanometer scale because of its cost-effectiveness and straightforward manufacturing process.

The TPU/PTUEG_3_ fabrics can be healed at room temperature by applying external force,^20^ even after repeated cycles. Additionally, their self-healing ability can be further enhanced through heating. The tensile tests and DSC analyses indicate that the mechanical properties and self-healing capabilities of the fabrics can be controlled by the PTUEG_3_ content. Optimal properties can be achieved at specific TPU/PTUEG_3_ weight ratios at which the fabrics maintain adequate stretchability while maximizing their healing efficiency. This study provides a feasible approach to efficiently and affordably produce intrinsically healable fabrics, which may also inspire future research to further advance the development of healable fabrics.

## Results and Discussion

**Figure 1:**
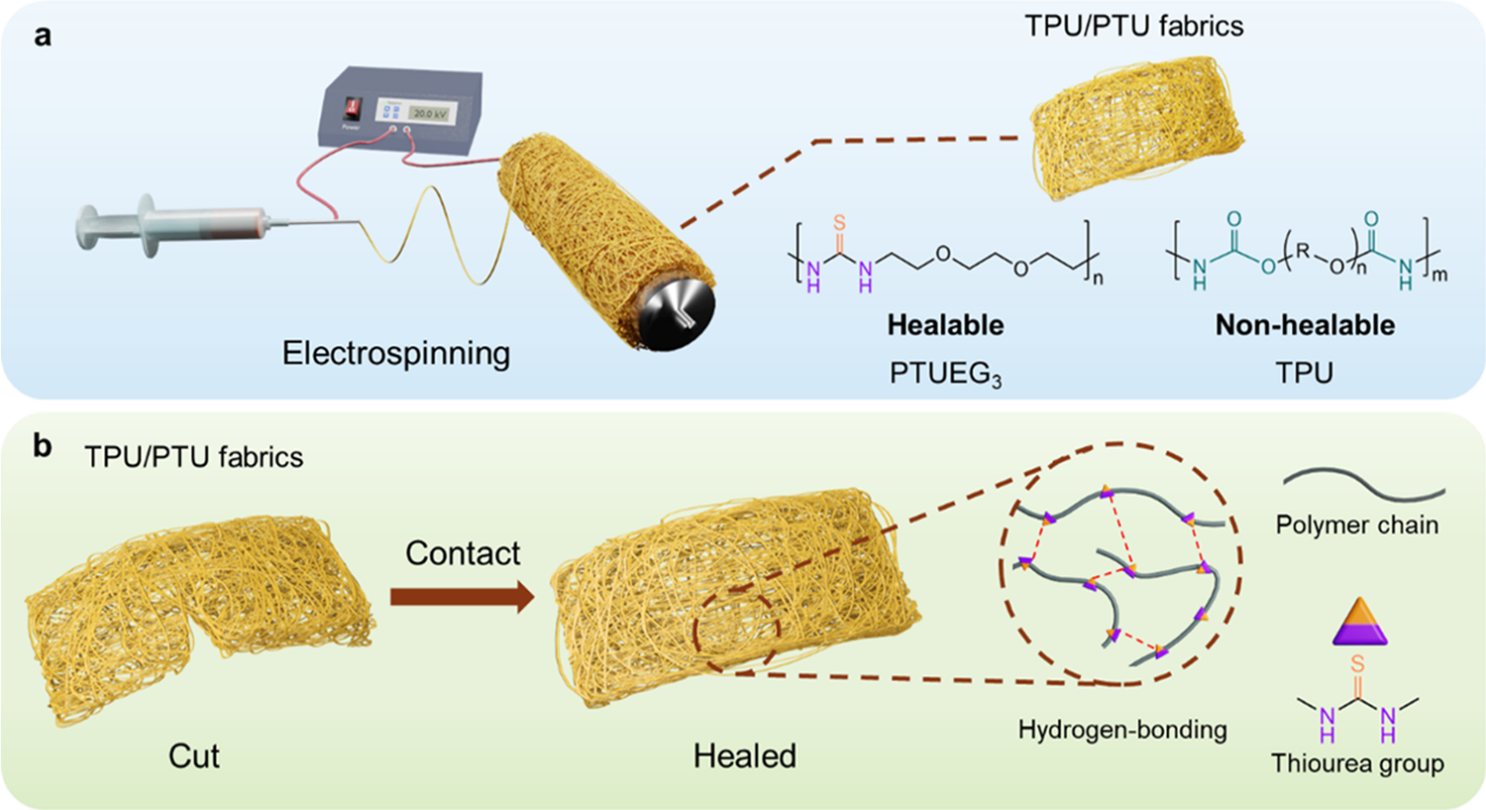
Fabrication and healing mechanism of the healable electrospun fabrics. (a) Fabrication of the healable TPU/PTUEG_3_ fabrics using the electrospinning method. (b) Schematic illustrations of the electrospun fibers with healable capabilities and the corresponding healing mechanism. The cut fabrics can be restored through a simple process of contact and pressing.

Figure 1 illustrates the experimental procedures involved in fabricating the healable electrospun fabrics and the healing mechanism. In this work, highly stretchable TPU is employed to impart stretchability, while intrinsically self-healable material PTUEG_3_ is synthesized and utilized for healing properties. In Figure 1a, TPU and synthesized PTUEG_3_ are dissolved in a cosolvent system comprising dimethylformamide (DMF) and tetrahydrofuran (THF) to form a polymer solution. The solution is transferred to a syringe and injected at a suitable rate and voltage during the electrospinning process. The rotating roller is used as a collector to obtain uniformly thick fabrics. As depicted in Figure 1b, when fabrics are cut, the polymer chains on the surface of the damaged areas diffuse and undergo restoration by forming new hydrogen bonds to repair the fabrics through overlapping and clamping of the damaged regions.

**Figure 2:**
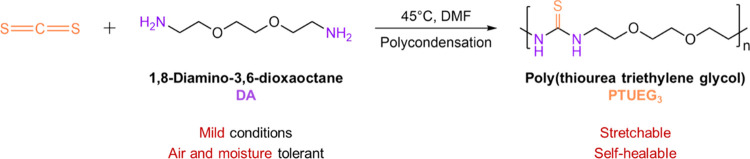
Synthetic scheme of the PTUEG_3_. The polycondensation of carbon disulfide and 1,8-diamino-3,6-dioxaoctane can occur under mild conditions.

The PTUEG_3_ used in this work is synthesized via a condensation polymerization, as illustrated in Figure 2. The chemical structure of the product is verified by the ^1^H nuclear magnetic resonance (NMR) spectrum, as demonstrated in Figure S1. The reaction occurs immediately and violently when two monomers are added together and can proceed in the absence of a catalyst. Air and moisture are not required to be removed because the reaction is less sensitive to them. Solvents are not necessary for this reaction, but in this study, all reactions are conducted in DMF, which is a good solvent for the product. This process is done to prevent the viscosity of the solution from increasing significantly and to avoid the reduction in the reaction rate and conversion. The decomposition temperature (*T*_d_) of the PTUEG_3_ at 5 wt % is confirmed through thermogravimetric analysis (TGA) to be 273 °C, as shown in Figure S3, demonstrating that the PTUEG_3_ exhibits good thermostability and is suitable for common applications.

The PTUEG_3_ is synthesized according to the procedures in the prior literature.^21^ The reaction conditions and their effects on the yield, molecular weight, and dispersity are summarized in Table 1. Prolonged reaction times (entries 1–2) significantly enhance both the yield and the weight-average molecular weight (*M*_w_). These results demonstrate that extended durations allow for more complete polymerization. However, excessively long reaction times may lead to energy inefficiencies. The effect of feed ratios between diamine (DA) and carbon disulfide (CS_2_) is also explored (entries 2–3). While the stoichiometric ratio of diamine to CS_2_ is 1:1, an excess of CS_2_ is employed to compensate for its low boiling point (∼45 °C) and the potential for evaporation during the reaction. When the feed ratio is set at 2:3 (entry 3), *M*_w_ increases significantly because of the role of CS_2_ in forming an isothiocyanate intermediate, facilitating effective polymerization. In contrast, a higher excess of CS_2_ (1:5, entry 1) results in incomplete polymerization, leading to lower *M*_w_ values and yields. Entry 3 is selected as the optimal condition, offering a favorable balance of high molecular weight and yield, which is critical for subsequent applications.

**Table 1:** Reaction Conditions for Polycondensation of Diamine and Carbon Disulfide

entry	DA/CS_2_ (molar ratio)	DA (mmol)	T (°C)	time (h)	yield of PTU (%)	*M*_w_ (kg/mol)	*Đ*
1	1:5	10	45	5	58.8	2.14	1.44
2		10	45	24	83.3	5.43	1.27
3	2:3	10	45	24	72.5	18.77	1.60

Because an excess amount of CS_2_ is used during the synthesis, ^13^C NMR spectroscopy is utilized to confirm the absence of residual CS_2_ in the final PTUEG_3_ product, as shown in Figure S2. The molecular characteristics of PTUEG_3_ are investigated using gel permeation chromatography (GPC), with the results summarized in Table 1 and Figure S4. For comparison, the weight-average molecular weight (*M*_w_) and dispersity (*Đ*) of the purchased TPU are measured and are presented in Figure S5. These analyses collectively verify the polymer’s purity, molecular weight distribution, and dispersity, ensuring its suitability for subsequent applications.

**Figure 3:**
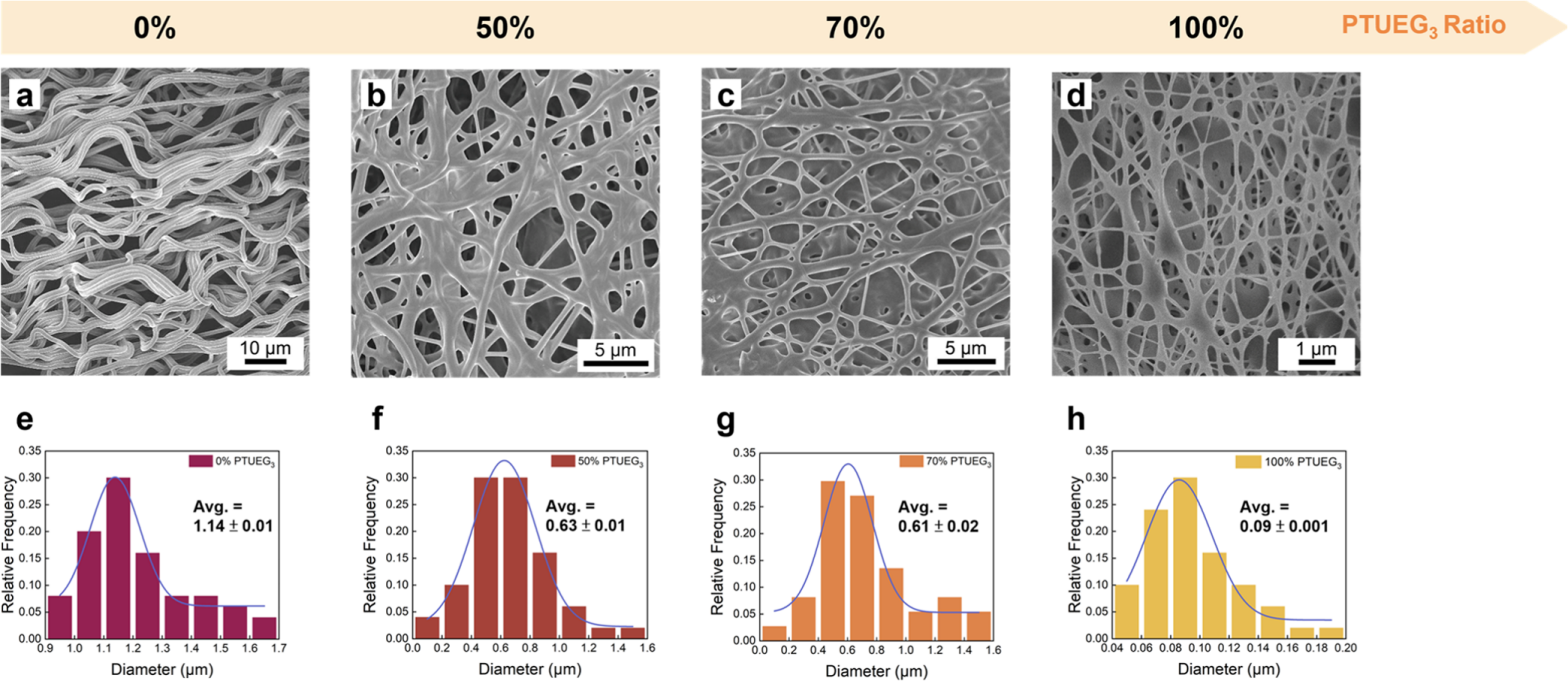
(a–d) SEM images of the TPU/PTUEG_3_ fabrics obtained using solutions with a DMF/THF ratio of 70:30 and different TPU/PTUEG_3_ weight ratios (100:0, 50:50, 30:70, and 0:100). (e–h) Corresponding diameter distributions of the TPU/PTUEG_3_ fibers shown in parts (a–d).

Figure 3 shows the morphologies of the TPU/PTUEG_3_ electrospun fibers with different TPU/PTUEG_3_ ratios. The TPU granules and synthesized PTUEG_3_ solid are dissolved in DMF/THF cosolvents under stirring for 12 h until fully dissolved to form a homogeneous solution. Then, the solutions are fabricated into fabrics using the electrospinning method and kept in a hood for at least 1 day to remove the residual solvent. The morphologies are investigated by scanning electron microscopy (SEM). The DMF in the cosolvent system is an effective solvent for TPU and PTUEG_3_ to form well-dissolved polymer solutions. DMF, however, exhibits very low volatility in the atmosphere, resulting in residue on the electrospun fibers and affecting the fibrous morphologies. In contrast, THF is a highly volatile solvent that evaporates rapidly during the electrospinning process. Adding THF to the solvent mixture can help reduce the residual solvent on the fabrics. The other DMF/THF ratios are not included here because the solubility of TPU/PTUEG_3_ decreases with the increasing amount of THF. The chosen ratio of DMF to THF in this study balances solubility and volatility to achieve the most suitable conditions for electrospinning.

Figure 3a,e shows the SEM image of the TPU fibers (TPU/PTUEG_3_ ratio of 100:0) and the corresponding diameter distributions. Before TPU was blended with PTUEG_3_, the diameters of the fibers were ∼1.14 μm. After increasing the PTUEG_3_ ratio to 50%, the average diameter decreases to ∼0.63 μm (Figure 3b,f). When the ratio of PTUEG_3_ is further increased to 70%, the average diameter does not decrease significantly, reducing to only ∼0.61 μm (Figure 3c,g). As the fibers consist entirely of PTUEG_3_ (TPU/PTUEG_3_ ratio 0:100), the average diameter decreases significantly to ∼0.09 μm (Figure 3d,h). The decreasing fiber diameter with increasing PTUEG_3_ contents highlights the impact of the PTUEG_3_ contents on solution viscosity, electrospinning behavior, and fiber formation. The tunable fiber diameters reflect compositional differences and play a critical role in determining the key material properties, including flexibility, mechanical strength, and self-healing efficiency.

The impact of density on the material properties is considered and calculated based on the mass and volume of the fabrics, as shown in Table S1. The density values for the 50 and 70% PTUEG_3_ conditions are relatively close to each other. For the 50% PTUEG_3_ condition, the densities range from 1.015 to 1.102 g/cm^3^, whereas for the 70% PTUEG_3_ condition, they range from 1.053 to 1.104 g/cm^3^. The minimal differences observed between the two conditions are attributed to the similar fiber diameters and fabric thicknesses, indicating that the PTUEG_3_ concentration does not significantly affect the overall density of the materials. This relatively small variation in density suggests that other factors, such as the polymer matrix composition and the fabrication process, may play a more prominent role in influencing the mechanical properties and self-healing behavior of the materials.

**Figure 4:**
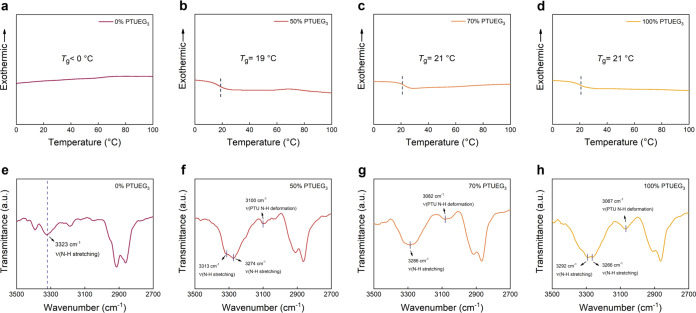
(a–d) Differential scanning calorimetry (DSC) thermograms of the TPU/PTUEG_3_ fabrics obtained using solutions with a DMF/THF ratio of 70:30 and different TPU/PTUEG_3_ weight ratios (100:0, 50:50, 30:70, and 0:100). (e–h) Corresponding FTIR spectra of the TPU/PTUEG_3_ fibers shown in parts (a–d).

Figure 4a–d illustrates the differential scanning calorimetry (DSC) thermograms of the TPU/PTUEG_3_ fabrics with various TPU/PTUEG_3_ weight ratios (100:0, 50:50, 30:70, and 0:100). Both TPU and PTUEG_3_ are confirmed to be amorphous polymers, as evidenced by the absence of crystallinity peaks within the temperature range of −20 to 200 °C at a heating rate of 5 °C min^–1^. Complete DSC thermograms are also provided in Figure S7 to further verify this result. The glass transition temperatures (*T*_g_) are determined from the second heating cycles in the DSC thermograms. TPU exhibits high stretchability at room temperature, with no detectable *T*_g_ observed within this range, indicating a *T*_g_ below −20 °C (Figure 4a). Additional DSC experiments with a slow scanning speed of 2.5 °C min^–1^ over a temperature range from −25 to 250 °C are also conducted, by which *T*_g_ is still not observed (Figure S8). The *T*_g_ values of the TPU/PTUEG_3_ blends are clearly observed in Figure 4b–d. With increasing PTUEG_3_ concentrations, the *T*_g_ values shift to higher temperatures due to stronger hydrogen bonding interactions: 19 °C for 50% PTUEG_3_ and 21 °C for both 70 and 100% PTUEG_3_. These hydrogen bonding interactions within PTUEG_3_ restrict the chain mobility, resulting in increased *T*_g_. Further confirmation of the amorphous nature of the materials is provided by XRD analysis (Figure S9), where broad peaks at 2θ angles of 21.3, 22, and 22.1° are for the 50:50, 30:70, and 0:100 TPU/PTUEG_3_ weight ratios, respectively, reflecting their noncrystalline structures. The self-healing performance of the TPU/PTUEG_3_ fabrics is intrinsically linked to their low crystallinity and *T*_g_ values. In hydrogen bonding-driven self-healing systems, the healing process requires polymer chain diffusion and the reformation of hydrogen bonds. Polymer chains in crystalline regions are immobile, making amorphous regions the main contributors to healing. Below *T*_g_, limited chain mobility restricts healing efficiency. Therefore, heating above *T*_g_ is essential for achieving effective self-healing. The moderate *T*_g_ values and low crystallinity of the TPU/PTUEG_3_ blends highlight their excellent potential for self-healing applications. The hydrogen bonding interactions within the TPU/PTUEG_3_ system are further investigated through FTIR analysis (Figure 4e–h). Vibrational bands around 3300 cm^–1^ correspond to the N–H stretching vibrations, a feature common to both TPU and PTUEG_3_. However, a distinct vibrational band within the range of 3100 to 3060 cm^–1^, corresponding to nonlinear hydrogen bond arrays, is observed exclusively in PTUEG_3_ and its blends but is absent in pure TPU (Figure 4e). This unique feature underscores the presence of dynamic and robust hydrogen bonding interactions introduced by the thiourea groups in PTUEG_3_. These findings confirm that the TPU/PTUEG_3_ blends possess an extensive hydrogen bonding network, which is critical for polymer chain diffusion and bond reformation during self-healing.

**Figure 5:**
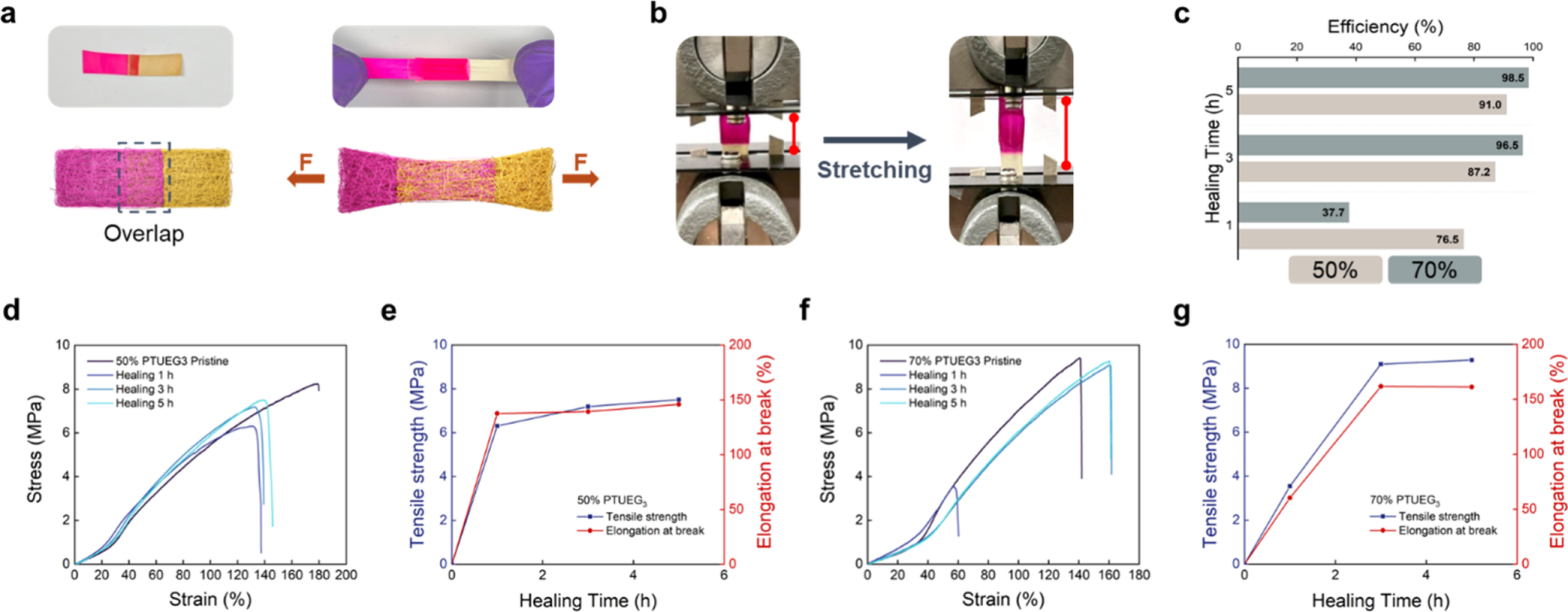
(a) Illustrations and corresponding photographs of lap-healing fabrics and stretched fabrics after healing. One of the cut fabrics is dyed with Rhodamine 6G for observation. (b) Photographs of the fabric before and during stretching in the tensile test analysis. (c) Relationship between efficiency and healing time of the TPU/PTUEG_3_ fabrics with different weight ratios (50:50 and 30:70). Pure TPU fabric (TPU/PTUEG_3_ ratio 100:0) and PTUEG_3_ fabric (TPU/PTUEG_3_ ratio 0:100) are not included because the former does not exhibit healing ability, and the latter has poor stretchability compared with the TPU-containing fabrics. (d, f) Stress–strain curves of the TPU/PTUEG_3_ fabrics with different weight ratios (50:50 and 30:70) under different healing times. (e, g) Plot of the tensile strength and elongation at break of the healed fabrics for different healing times corresponding to parts (d, f).

The healing process is demonstrated in Figure 5a, where a rectangular fabric is cut into two pieces.

To facilitate clear observation, one of the cut fabrics is dyed magenta with Rhodamine 6G. Subsequently, the two fabrics are overlapped in a small area at the end of the cut region and sandwiched between PTFE sheets and acrylic plates. Binder clips are used to apply compressive forces, and the fabrics are heated to 50 °C for a specified period. This method simulates real-world scenarios where localized damage occurs in fibrous systems. It is important to note that healing does not occur upon simple contact, as compressive forces are necessary to facilitate molecular diffusion and hydrogen bond reformation at the damaged interface, ensuring effective recovery. The tensile strength of the healed fabrics is compared with that of the pristine fabrics to assess healing efficiency. The tensile properties and healing efficiency of the fabrics are studied through tensile test analysis. Figure 5b displays photographs of the tensile test analysis. The fabrics are cut into two pieces measuring 1 × 1.75 cm^2^ each, with the overlap region set at 1 × 0.5 cm^2^. As shown in Figure 5c, the healing efficiency improves significantly with longer healing times, recovering to over 90% for both 50 and 70% PTUEG_3_ fabrics after at least 3 h of healing. The 70% PTUEG3 blend achieves a nearly complete healing efficiency (∼98.5%) within 5 h, while the 50% PTUEG_3_ blend reaches ∼91% under the same conditions. It is important to note that healing does not occur upon simple contact. Instead, compressive forces are essential to facilitate molecular diffusion and hydrogen bond reformation at the damaged interface, ensuring targeted repair rather than unintended adhesion.

To further validate the ability of the TPU/PTUEG_3_ system for repeated self-healing, scanning electron microscopy (SEM) images of the healed regions (Figure S10) demonstrate that the fibrous structure remains intact after the healing process, with only minor morphological differences compared with the pristine state. Retaining fiber structure following compression and heating treatment supports the material’s potential for repeated self-healing applications. In addition, energy-dispersive X-ray spectroscopy (EDS) and X-ray photoelectron spectroscopy (XPS) analyses (Figures S11 and S12) confirm the uniform distribution of PTUEG_3_ within the fibers, ensuring a consistent performance throughout the textile system. These characterizations collectively validate the uniform composition and structural integrity of the TPU/PTUEG_3_ fabrics, highlighting their suitability for practical applications in which localized damage and repair are more representative of real-world use cases.

To investigate the role of the PTUEG_3_ concentration in the hydrogen bonding strength and its influence on self-healing, XPS analyses are performed on TPU/PTUEG_3_ blends with 50, 70, and 100% PTUEG_3_ (Figures S13–S15). The C 1s, N 1s, O 1s, and S 2p spectra exhibit minimal shifts in binding energy and peak intensity, indicating that the intrinsic hydrogen bonding environment remains consistent across different PTUEG_3_ concentrations. This observation suggests that variations in the PTUEG_3_ content primarily affect molecular mobility and overall composition rather than altering the fundamental strength of hydrogen bonds. Such consistency is critical for enabling sustained self-healing performance and ensuring that repeated healing cycles do not degrade the intrinsic bonding properties of the material.

To evaluate the mechanical recovery process, stress–strain curves of fabrics healed for different durations are presented in Figure 5d and 5f, illustrating the time-dependent restoration of mechanical properties. The tensile strength and elongation at break as functions of the healing time are summarized in Figure 5e and g. For the 50% PTUEG_3_ fabrics, the tensile strengths are recorded as 8.2 (pristine), 6.3 (1 h), 7.2 (3 h), and 7.5 MPa (5 h). For the 70% PTUEG_3_ fabrics, the tensile strengths are measured as 9.4 (pristine), 3.5 (1 h), 9.1 (3 h), and 9.3 MPa (5 h). These results demonstrate that the tensile strength recovers to near-pristine levels over time, particularly in the 70% PTUEG_3_ system, which exhibits faster and more efficient healing because of the higher concentration of hydrogen bonding interactions. The elongation at break follows a similar trend, with both systems reaching ∼150% or higher strain under suitable healing conditions. These results confirm the ability of the TPU/PTUEG_3_ system to effectively restore mechanical properties after damage, meeting the demands of applications requiring flexibility and durability.

To further understand the relationship between mechanical properties and self-healing behavior, the toughness of the materials, representing the energy absorbed before failure, is analyzed in detail (Table S2). The Young’s modulus, derived from the initial slope of the stress–strain curve, decreases with increasing PTUEG_3_ content, reflecting enhanced polymer flexibility and chain mobility. This reduction in stiffness is attributed to the higher density of hydrogen bonding interactions, which not only improve chain mobility but also facilitate the reformation of bonds during the healing process. Such flexibility is critical for effective self-healing, allowing the polymer chains to reconnect and repair damaged areas efficiently.

The 50% PTUEG_3_ fabric exhibits higher toughness because of the contribution of TPU, which is renowned for its mechanical strength and toughness. In contrast, the 70% PTUEG_3_ fabric demonstrates posthealing toughness that exceeds the pristine state. This improvement is attributed to the overlap region forming a thicker healed area, which enhances structural integrity during tensile testing. The increased thickness compensates for any localized weakness, contributing to the observed enhancement in toughness. These findings highlight the pivotal role of polymer composition and structural factors in influencing both the mechanical performance and the self-healing behavior of the TPU/PTUEG_3_ system. Optimizing the PTUEG_3_ content enables a desirable balance between stiffness, flexibility, and healing efficiency, paving the way for advanced applications in wearable electronics, flexible devices, and other technologies that require both durability and self-repairing capabilities.

## Conclusions

We developed stretchable, intrinsically healable fabrics based on TPU and PTUEG_3_. Our work highlights the successful application of electrospinning to produce these fabrics, which not only demonstrate healability at room temperature but also show enhanced self-healing properties when subjected to external heating. The robust hydrogen bonding interactions within the TPU/PTUEG_3_ blends facilitate mechanical recovery, ensuring consistent performance and durability, which underscores the potential of this technology to reduce the environmental impact by prolonging the lifespan of textile products. The findings from our tensile tests and DSC analyses indicate that the mechanical properties and self-healing capabilities of the fabrics are highly dependent on the PTUEG_3_ content, with an optimal balance achieved at specific TPU/PTUEG_3_ weight ratios. These ratios ensure that the fabrics maintain adequate stretchability while maximizing their healing efficiency. Our work demonstrates that the intrinsic self-healing mechanism driven by noncovalent hydrogen bonding is not only feasible but also efficient, offering a practical approach to the design of sustainable materials.

## Experimental Section

### Materials

1,8-Diamino-3,6-dioxaoctane was purchased from Nova Materials. Carbon disulfide was obtained from Honeywell. Thermoplastic polyurethane (TPU, 1685A-E2) was purchased from Great Eastern Resins Industrial Co. Ltd. The weight-average molecular weight (*M*_w_), number-average molecular weight (*M*_n_), and dispersity (*Đ*) of TPU are approximately 110, 49, and 2.2 kg/mol, respectively, as determined by GPC (Figure S5). The soft segment structure of the TPU is polyether-based (Figure S6), contributing to its flexibility and mechanical adaptability. Dimethylformamide (DMF, 99.8%) and methanol (MeOH, 99.5%) were procured from Thermo Scientific and Echo Chemical Co. Ltd., respectively. Rhodamine 6G was obtained from Sigma-Aldrich.

### Synthesis of Poly(thiourea triethylene glycol) (PTUEG_3_)

1,8-Diamino-3,6-dioxaoctane (1.459 mL, 10 mmol) was dissolved in DMF (1.5 mL) in a double-necked flask. After complete dissolution, carbon disulfide (CS_2_) was injected into the solution in varying amounts, as detailed in Table 1. The mixture was refluxed at 45 °C and stirred for 24 h. To safely absorb the toxic gaseous byproduct (H_2_S), the flask was connected to a beaker containing a NaOH aqueous solution. After completion, the reaction mixture was poured into a DMF/MeOH (50 mL) solution to induce precipitation. The resulting product was heated at 150 °C in a vacuum oven for 24 h to remove the residual solvent, yielding the final product as a yellow glass.

### Electrospinning Process of the TPU/PTU Blend Fibers

Initially, TPU granules and PTUEG_3_ were dissolved in DMF/THF (70:30 and 50:50) at a concentration of 30 wt % with various ratios of TPU and PTUEG_3_ (100:0, 70:30, and 50:50) using Parafilm-sealed glass bottles. Subsequently, each solution was transferred to a 5 mL disposable plastic syringe and connected with a stainless steel capillary needle, which was then placed on a syringe pump (KD Scientific). The flow rate was set at 1.5 mL/h, and the solution was ensured to flow smoothly before commencing. A 10 cm diameter roller, covered with baking paper and rotated at 600 rpm, functioned as the collector. The distance between the needle and the collector was 15 cm. The needle was connected to the positive pole of the power supply (SMICO), and the roller collector was grounded. A 20 kV voltage was provided by the power supply to create a Taylor cone during the electrospinning process. The fibers were collected for 8 h and then stored for subsequent use.

### Mechanical Strength of the TPU/PTUEG_3_ Blend Fibers

First, 1 × 3 cm^2^ pieces of TPU/PTUEG_3_ fabrics were prepared. Then, the TPU/PTUEG_3_ fabrics were loaded onto a tensile test machine by using a fixture for mechanical property measurements. The tensile test was performed under displacement control at room temperature with a 5 mm·min^–1^ displacement rate for uniaxial elongation.

### Structure Analysis and Characterization

The chemical structure of PTUEG_3_ was analyzed using a ^1^H nuclear magnetic resonance (NMR) spectrometer with a frequency of 400 MHz (Varian). Size exclusion chromatography (SEC) was conducted using a refractive index detector (JASCO RI-4030) and an autosampler (JASCO AS-4050) to determine the apparent weight-average molecular weight (*M*_w_), employing narrowly distributed polystyrene (PS) standards. Dimethylformamide (DMF) served as the mobile phase, with a flow rate maintained at 0.1 mL/min. Thermal analyses of the TPU and PTUEG_3_ were carried out employing a thermogravimetric analyzer (TGA, TA 55, TA Instruments). The samples were heated to 700 °C under a nitrogen atmosphere at a rate of 20 °C·min^–1^. The glass transition temperatures of both TPU and PTUEG_3_ were investigated by differential scanning calorimetry (DSC, TA Q200, TA Instruments). DSC measurements were performed under a nitrogen atmosphere in the temperature range of −20 to 200 °C, employing heating and cooling rates set at 5 °C·min^–1^. The morphologies of the TPU/PTUEG_3_ fabrics were characterized using a field-emission scanning electron microscope (SEM, JEOL JSM-7401F) with an accelerating voltage of 5 kV. The fabrics underwent drying in vacuum desiccators and were coated with a thin layer (∼4 nm) of platinum using a sputter coater (JEOL JFC-1600) with a 20 mA current for 50 s to enhance the conductivity. The ImageJ software was employed to determine the average diameter of the fibers (∼100 pieces) from the SEM images. The mechanical properties of the TPU/PTUEG_3_ fabrics were examined by using a uniaxial elongation mode on a tensile testing machine (Shimadzu EZ Test).

## Supporting Information Available

^1^H NMR spectra; ^13^C NMR spectra; TGA curve; GPC curves of synthesized poly(thiourea triethylene glycol) (PTUEG_3_); GPC curves of purchased TPU; table of density measurements of TPU/PTUEG_3_ fabrics; soft segment structure of the TPU; DSC thermograms of TPU/PTUEG_3_ fabrics with different TPU/PTUEG_3_ weight ratios and slower scanning speed; XRD profiles of PTUEG_3_ fabrics; SEM image of the overlap region after healing; EDS and XPS mapping analyses of TPU/PTUEG_3_ fabrics; XPS spectra of TPU/PTUEG_3_ fabrics at various weight ratios; and table of the mechanical properties of TPU/PTUEG_3_ fabrics (PDF)
