# Supplementary material for: Intrinsic Repeated Self-Healing Textiles: Developing Electrospun Fabrics for Enhanced Durability and Stretchability
Source: ACS Omega. 2024 Dec 16;9(52):51623–30. doi: 10.1021/acsomega.4c09296 (PMC11696384; doi:10.1021/acsomega.4c09296)
Supplement: Supplementary file 1 — ao4c09296_si_001.pdf [file ao4c09296_si_001.pdf]

Supporting Information

Intrinsic Repeated Self-Healing Textiles: Developing Electrospun Fabrics for Enhanced Durability and Stretchability

Tse-Yu Lo,[1] Heng-Hsuan Su,[1] Jhih-Hao Ho,[1,2] Chia-Wei Chang,[1] Huan-Ru Chen,[1] Hsun-Hao Hsu,[1] Kai-Jie Chang,[1] and Jiun-Tai Chen[1,3]*

[1]Department of Applied Chemistry, National Yang Ming Chiao Tung University, Hsinchu, Taiwan 300093

[2]Department of Chemistry, Duke University, Durham, NC, 27708, United States

[3]Center for Emergent Functional Matter Science, National Yang Ming Chiao Tung University, Hsinchu, Taiwan 300093

*To whom correspondence should be addressed. E-mail: jtchen@nycu.edu.tw.

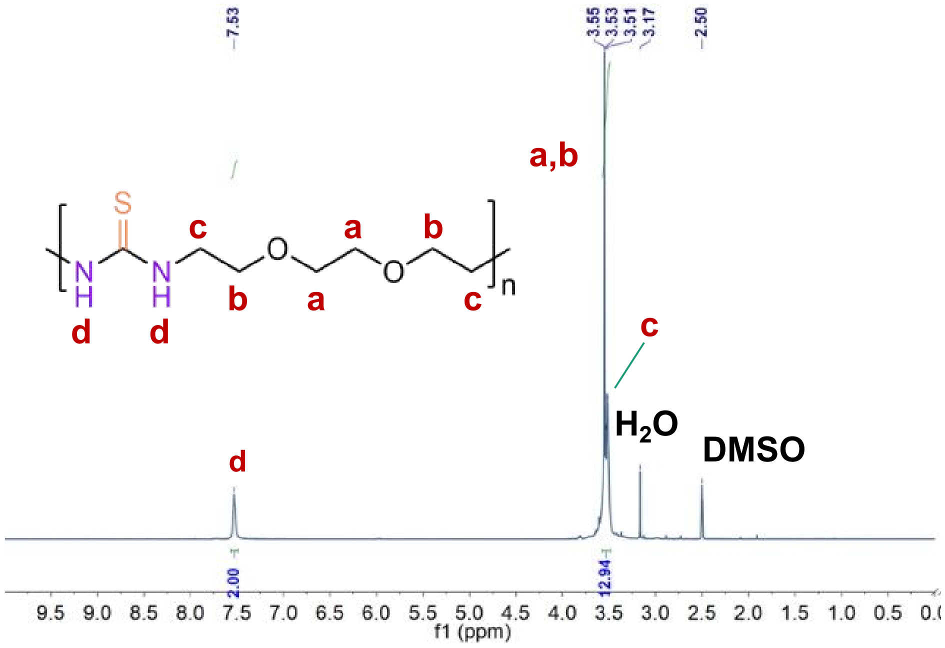

Figure S1. ^{1}H-NMR spectrum of $PTUEG_3$.

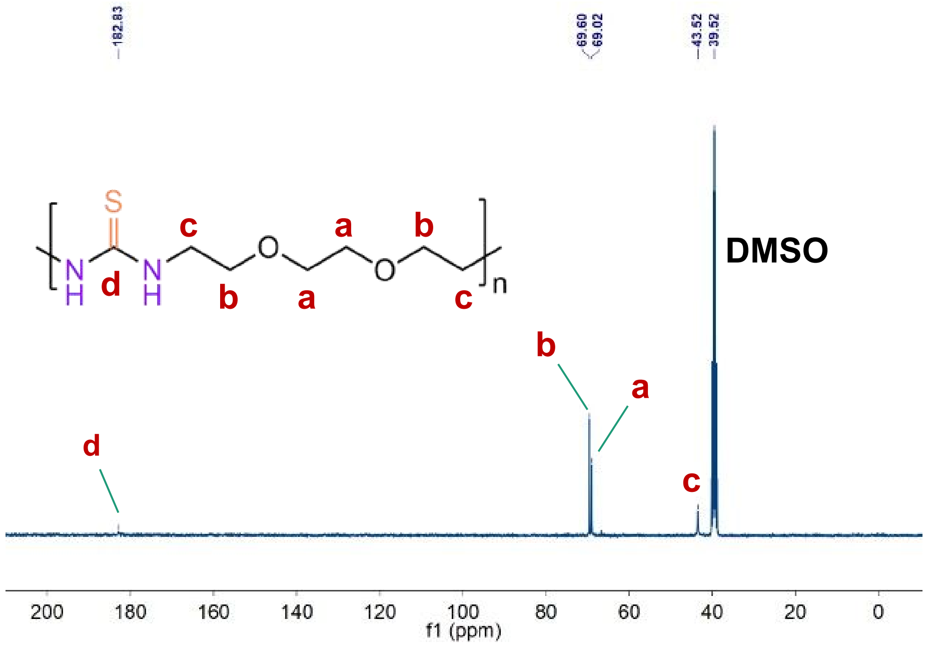

Figure S2. ^{13}C-NMR spectrum of $PTUEG_3$.

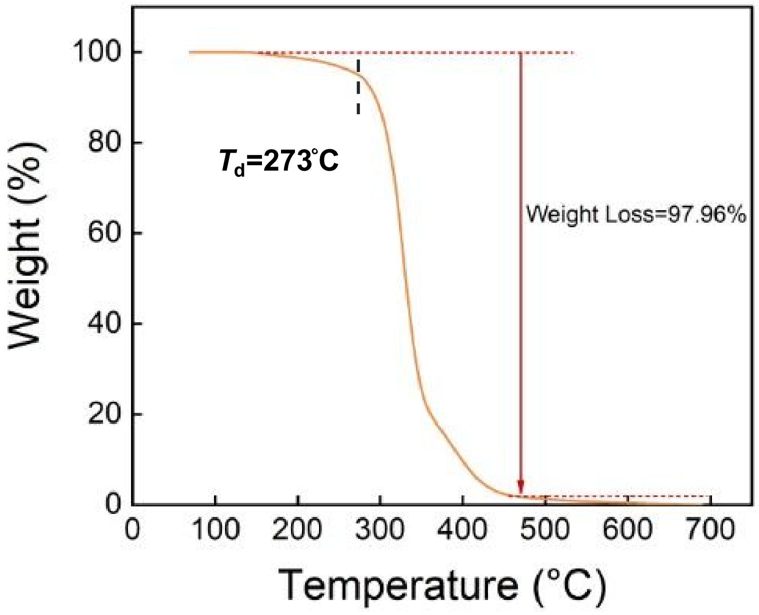

Figure S3. TGA curve of PTUEG$_3$.

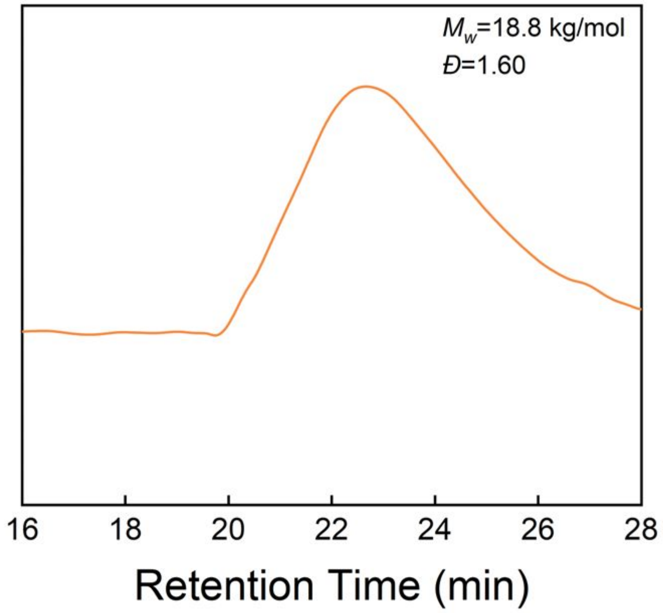

Figure S4. The GPC curve of PTUEG$_3$ from entry 3 in Table 1.

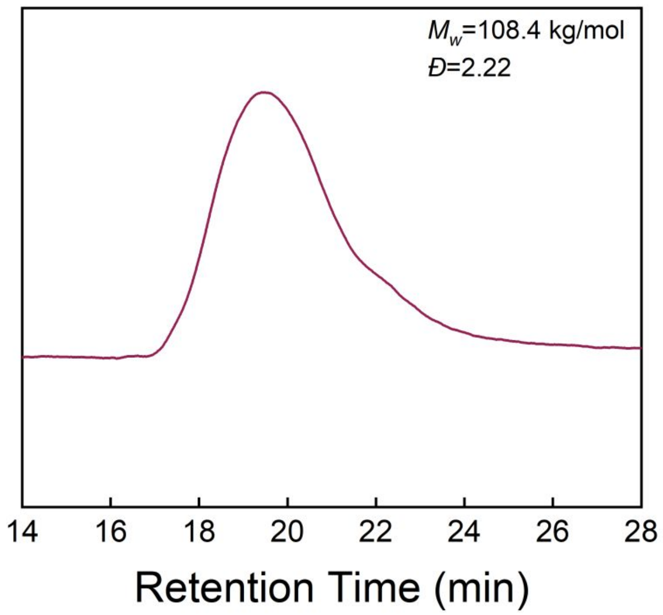

Figure S5. The GPC curve of TPU.

Table S1. Density of the TPU/$PTUEG_3$ fabrics

$PTUEG_3$ Ratio (%)	Weight (mg)	Thickness (mm)	Volume (cm^3)	Density (g/cm^3)
50	20.5	0.062	0.019	1.102
	19.8	0.065	0.020	1.015
	20.1	0.064	0.019	1.047
70	21.8	0.069	0.021	1.053
	22.8	0.071	0.021	1.070
	24.5	0.074	0.011	1.104

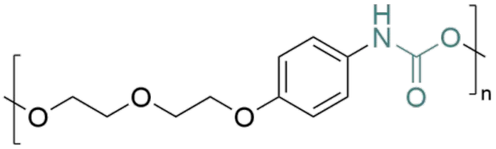

Figure S6. The soft segment structure of the TPU.

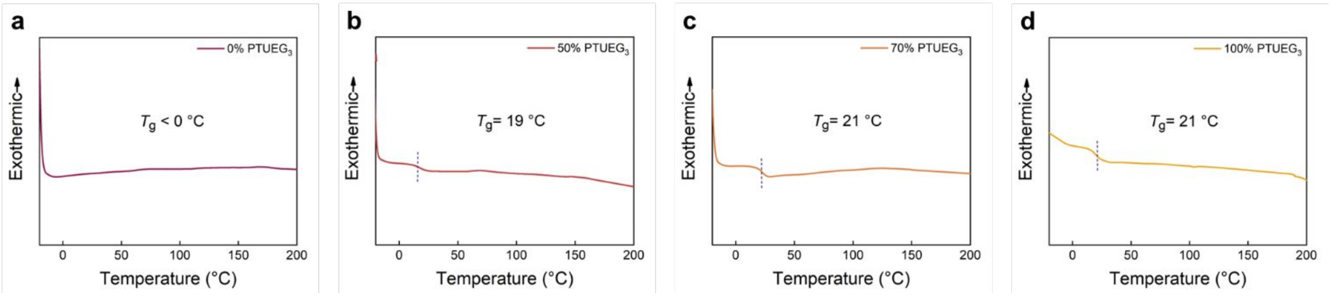

Figure S7. (a–d) DSC thermograms of TPU/PTUEG$_3$ fabrics with different TPU/PTUEG$_3$ weight ratios (100:0, 50:50, 30: 70, and 0:100).

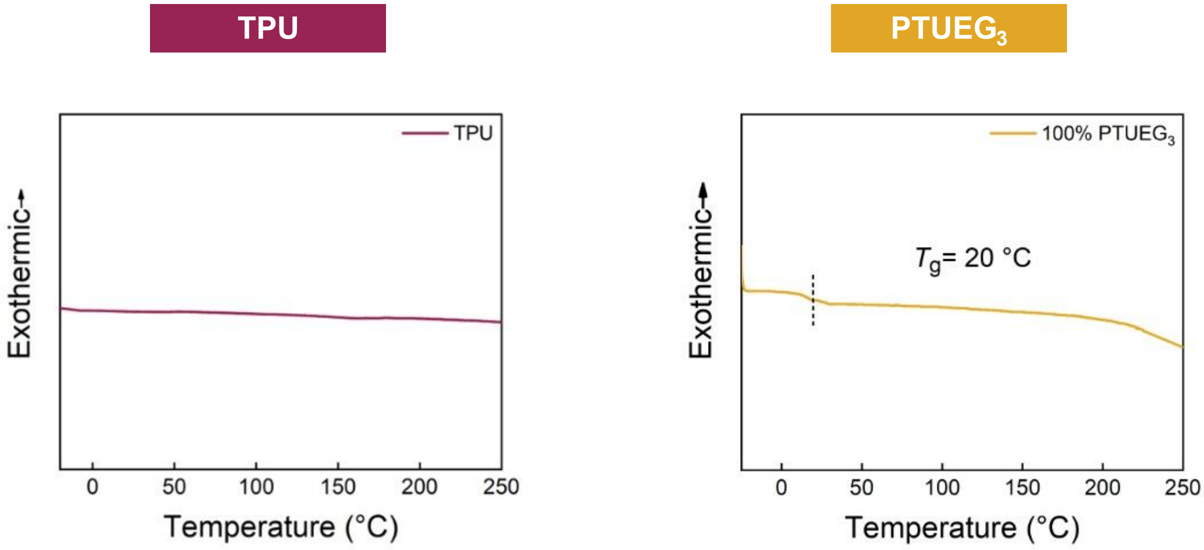

Figure S8. DSC thermograms of TPU and PTUEG$_3$ fabrics with slower scanning speed of 2.5 °C min^{-1}.

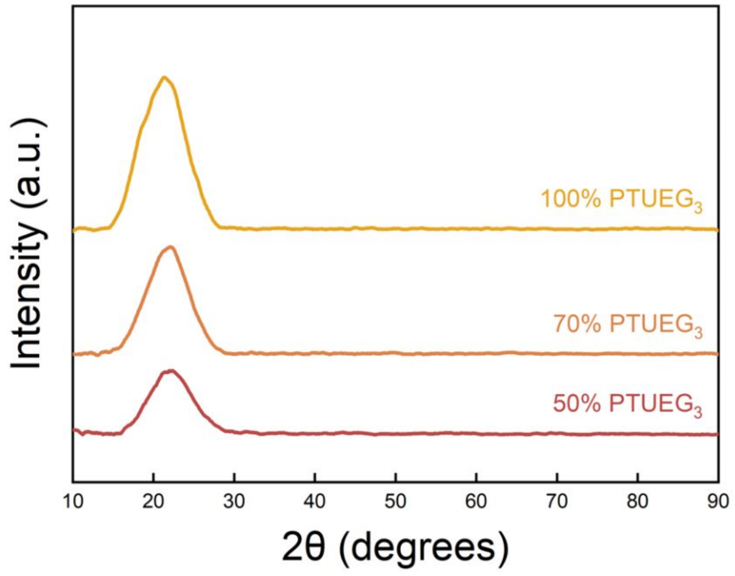

Figure S9. XRD profiles of 50, 70, and 100% $PTUEG_3$.

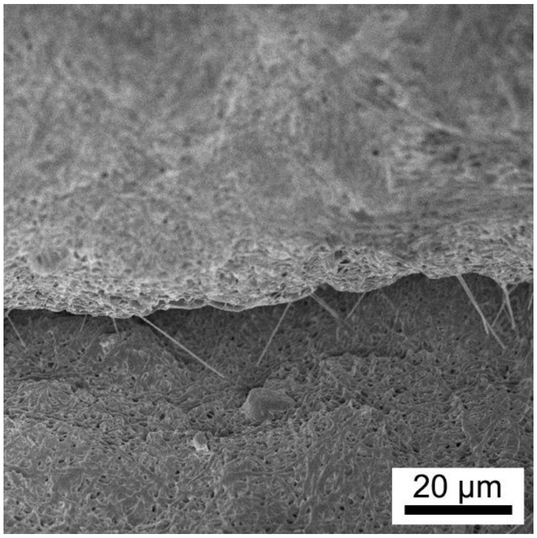

Figure S10. SEM image of overlap region after healing.

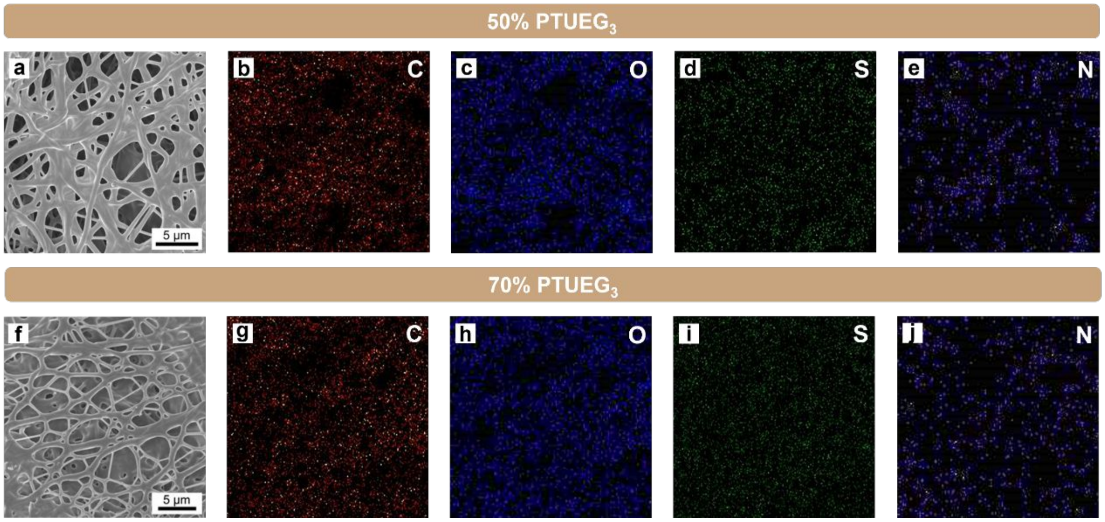

Figure S11. EDS mapping analysis of the elemental compositions in the 50% and 70% TPU/PTUEG$_3$ fabrics. (a, f) SEM images of the fibers. (b–e, g–j) Elemental mapping of C, O, S, and N corresponding to (a, f).

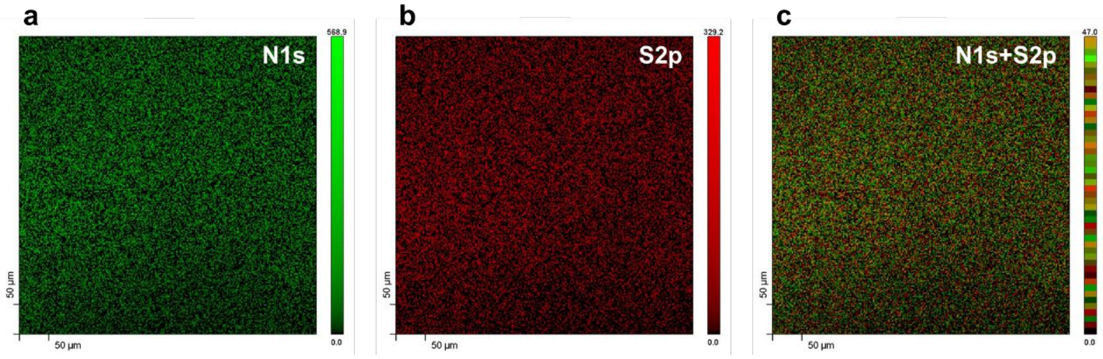

Figure S12. XPS mapping analysis of the elemental compositions in the TPU/PTUEG$_3$ fabrics. (a–c) Nitrogen (N1s), sulfur (S2p), and combined mapping of N1s and S2p.

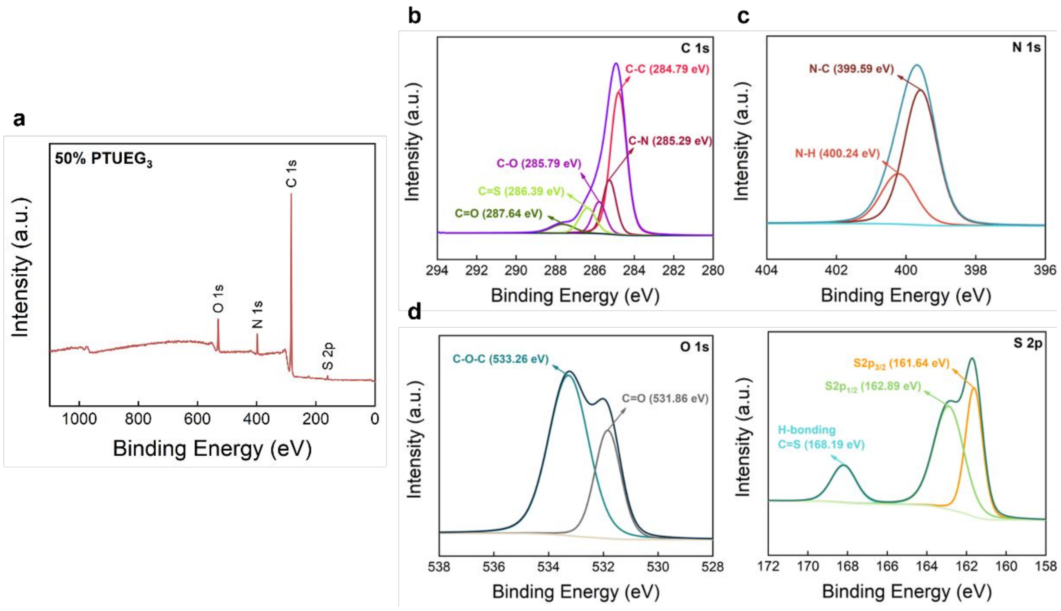

Figure S13. XPS spectra of the elemental compositions in the TPU/PTUEG$_3$ fabrics with a 50:50 weight ratio. (a) Full survey spectrum. (b–e) C1s, N1s, O1s, and S2p spectra.

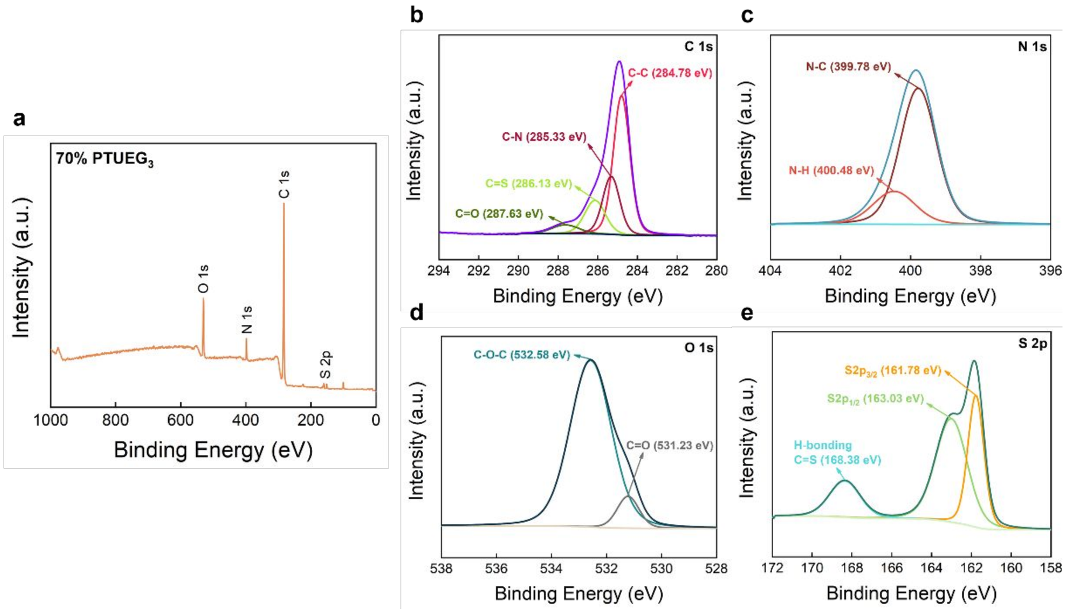

Figure S14. XPS spectra of the elemental compositions in the TPU/PTUEG$_3$ fabrics with a 30:70 weight ratio. (a) Full survey spectrum. (b–e) C1s, N1s, O1s, and S2p spectra.

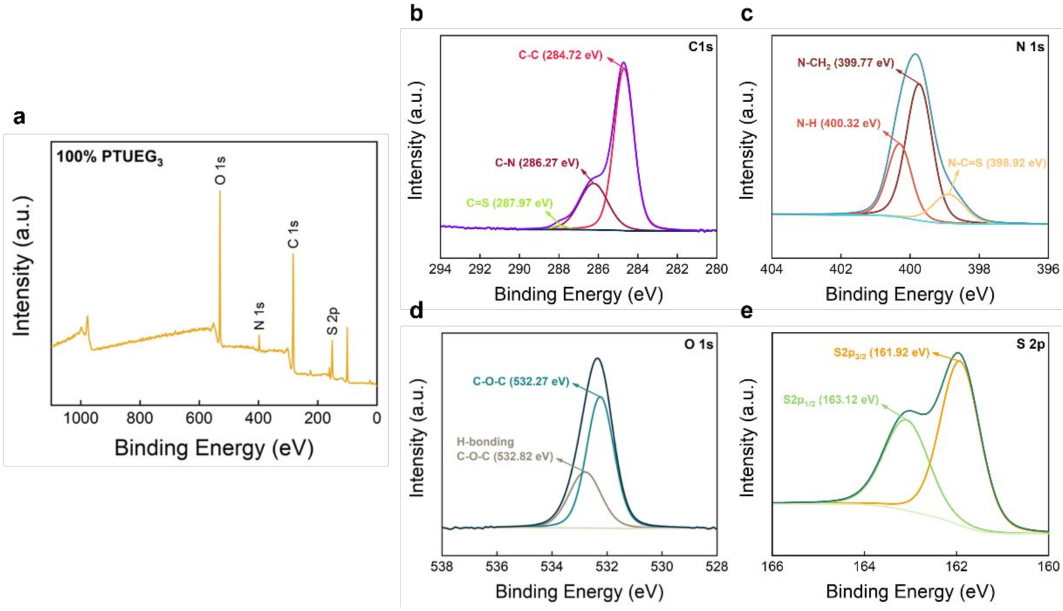

Figure S15. XPS spectra of the elemental compositions in the TPU/$PTUEG_3$ fabrics with a 0:100 weight ratio. (a) Full survey spectrum. (b–e) C1s, N1s, O1s, and S2p spectra.

Table S2. Mechanical properties of the TPU/$PTUEG_3$ fabrics

Healing Time (h)	$PTUEG_3$ Ratio (%)	Tensile Strength (MPa)	Elongation at Break (%)	Young's modulus (MPa)	Toughness (MJ/m^3)
Pristine	50	8.2	179.8	0.029	816.7
	70	9.4	141.9	0.026	646.3
1	50	6.3	137.5	0.040	503.6
	70	3.5	60.3	0.037	87.4
3	50	7.1	139.2	0.036	549.4
	70	9.1	161.7	0.028	715.2
5	50	7.5	145.8	0.034	582.7
	70	9.3	161.2	0.027	723.3
